# Dasiglucagon for the Treatment of Congenital Hyperinsulinism: A Randomized Phase 3 Trial in Infants and Children

**DOI:** 10.1210/clinem/dgad648

**Published:** 2023-11-01

**Authors:** Paul S Thornton, Diva D De Leon, Susann Empting, David Zangen, David M Kendall, Sune Birch, Eva Bøge, Jelena Ivkovic, Indraneel Banerjee

**Affiliations:** Congenital Hyperinsulinism Center, Cook Children’s Medical Center, Fort Worth, TX 76104, USA; Congenital Hyperinsulinism Center, Division of Endocrinology and Diabetes, Children’s Hospital of Philadelphia, Philadelphia, PA 19104, USA; Department of Pediatrics, Perelman School of Medicine at the University of Pennsylvania, Philadelphia, PA 19104, USA; Department of Pediatrics, Otto-von-Guericke University, Magdeburg 39120, Germany; Division of Pediatric Endocrinology, Hadassah Medical Center, Faculty of Medicine, Hebrew University of Jerusalem, Jerusalem 91240, Israel; Research and Development, Zealand Pharma A/S, Søborg 2860, Denmark; Research and Development, Zealand Pharma A/S, Søborg 2860, Denmark; Research and Development, Zealand Pharma A/S, Søborg 2860, Denmark; Research and Development, Zealand Pharma A/S, Søborg 2860, Denmark; Department of Paediatric Endocrinology, Royal Manchester Children's Hospital, Manchester M13 9WL, UK

**Keywords:** congenital hyperinsulinism, hypoglycemia, treatment, dasiglucagon

## Abstract

**Context:**

Congenital hyperinsulinism (CHI) is characterized by dysregulated insulin secretion causing hypoglycemia and consequent brain damage. Dasiglucagon is a glucagon analogue under investigation to treat CHI.

**Objective:**

To evaluate the efficacy and safety of dasiglucagon delivered via continuous subcutaneous infusion to children with CHI and persistent hypoglycemia as add-on to standard of care (SoC).

**Methods:**

In this open-label trial, patients were randomized 1:1 to SoC or SoC + dasiglucagon (10-70 µg/h) for 4 weeks. In the following 4 weeks, all patients received dasiglucagon + SoC. Hypoglycemia was assessed by self-monitored plasma glucose (SMPG) and blinded continuous glucose monitoring (CGM). Primary endpoint was average number of SMPG-detected hypoglycemia episodes/week (SMPG <3.9 mmol/L) during Weeks 2 to 4.

**Results:**

Thirty-two patients (0.6-10.9 years) were randomly assigned to dasiglucagon + SoC (n = 16) or SoC (n = 16). The rate of SMPG-detected hypoglycemia decreased from baseline in both groups, but with no statistically significant difference during Weeks 2 to 4 (event rate ratio: 0.85 [0.54; 1.36], *P* = .5028). However, dasiglucagon administration resulted in a 43% reduction in CGM-detected hypoglycemia (<3.9 mmol/L) vs SoC alone during Weeks 2 to 4 (post hoc analysis; event rate ratio: 0.57 [0.39; 0.83], *P* = .0029). Dasiglucagon enabled reductions (of 37% to 61%) in all other measures of hypoglycemia assessed by CGM vs SoC alone including extent and percent time in hypoglycemia (post hoc analyses). Dasiglucagon appeared safe and well tolerated. Skin and gastrointestinal events were more frequent with dasiglucagon + SoC than SoC only.

**Conclusion:**

Clinically meaningful reductions in all CGM-recorded measures of hypoglycemia support using dasiglucagon as a potential treatment for CHI.

Congenital hyperinsulinism (CHI) is a rare condition with an estimated incidence ranging from approximately 1:2500 to 1:50 000 (1). In CHI, pancreatic β-cells secrete insulin irrespective of blood glucose concentration (1, 2). This results in persistent and often severe hypoglycemia in neonates and early childhood, which can lead to brain damage, seizures, and neurodevelopmental impairment in the absence of timely diagnosis and effective treatment (1).

CHI can be acquired or genetic, with both forms associated with an adverse impact on neurodevelopment in up to 50% of patients (3, 4). Other forms of neonatal hyperinsulinism tend to be transient in nature and are often associated with perinatal stress or maternal gestational diabetes (1). The genetic forms can be single gene defects with symptoms related predominately to hypoglycemia secondary to hyperinsulinism, or they may occur as part of a syndrome in which hypoglycemia secondary to hyperinsulinism is only one manifestation of the syndrome. To date, a number of genes have been identified that cause hyperinsulinism, with many involved in the insulin secretory pathway (5). The genetic forms of CHI are generally classified as either focal, diffuse, or atypical according to histology (1). Current treatments for CHI include pharmacotherapy, nutritional support, and surgical management. At present, the potassium channel activator diazoxide is the only approved drug for the treatment of hyperinsulinism. However, this drug is ineffective in patients with inactivating mutations in the ATP-sensitive potassium channel, who represent the largest proportion of patients with known genetic cause (5). Several drugs not approved for use in the treatment of CHI have been utilized: mostly somatostatin analogues (octreotide and lanreotide), but also calcium channel blockers, mTOR inhibitors (sirolimus), and reconstituted glucagon (1, 5). However, a sizable proportion of children with CHI do not respond sufficiently to existing pharmacotherapies. Consequently, many patients continue to experience hypoglycemia or require nutritional support, such as continuous feeding through a nasogastric or gastric tube to prevent hypoglycemia. In a proportion of patients with diffuse or atypical CHI, medical and nutritional management is inadequate to ensure satisfactory glycemic stability. In these cases, subtotal or near-total pancreatectomy needs to be undertaken to reduce the frequency of hypoglycemia. However, such procedures are complicated by the risk of persistent hypoglycemia in 40% to 60% of patients (6, 7), the need for pancreatic enzyme replacements, and inevitable progression to insulin-requiring diabetes. By contrast, in focal CHI, most patients undergoing lesionectomy of the pancreatic lesion are cured (8). However, focal lesions in the head of the pancreas abutting on the bile duct may not be amenable to lesionectomy and require extensive surgical maneuvers such as pancreatojejunostomy.

Reconstituted glucagon is not approved for use in CHI but has been shown to be effective in the treatment of CHI (9, 10). The glycogenolytic effect of glucagon and its ability to increase plasma glucose levels has been confirmed in children with CHI (11, 12), with transient administration of glucagon (via intravenous [IV] continuous infusion or as bolus subcutaneous [SC] or intramuscular injections) often used during or after diagnosis to stabilize CHI patients before initiation of other medical or surgical treatments. While IV glucagon is used in the hospital setting (eg, before pancreatectomy), this treatment is generally limited to short-term use due to technical issues and the occurrence of significant gastrointestinal side effects (8, 13, 14). There are currently no marketed glucagon products available for long-term home use.

Dasiglucagon is a novel glucagon analogue under development for the prevention and treatment of hypoglycemia in patients with CHI. Dasiglucagon is currently approved as a rescue treatment for severe hypoglycemia in pediatric and adult patients with diabetes (15). Like human glucagon, dasiglucagon comprises 29 amino acids, with 7 amino acid substitutions introduced to improve stability in aqueous media, enabling dasiglucagon to be formulated as a ready-to-use aqueous solution deliverable via SC injection or SC infusion (16). In a preliminary report from a randomized, controlled phase 3 trial in neonates and infants with CHI (aged 7 days to 12 months), dasiglucagon significantly reduced the requirement for IV glucose to maintain glycaemia by 55% compared to placebo (17).

Here we report the results of a phase 3 trial that investigated the efficacy and safety of dasiglucagon delivered via a SC infusion pump on rates of hypoglycemia in infants and children with CHI (aged 3 months to 12 years) when added to standard of care (SoC) therapies.

## Methods

### Study Design

**Figure 1.**
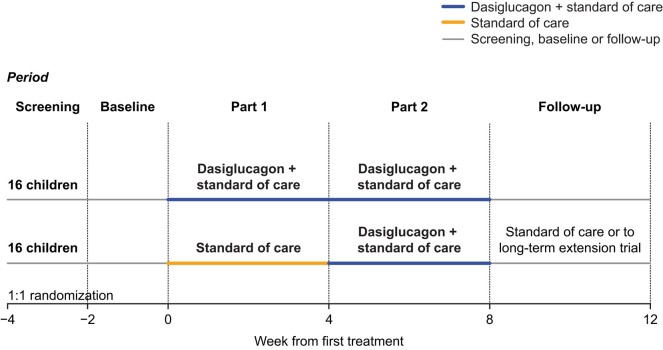
Trial design.

This 2-part, open-label, randomized trial evaluating the efficacy and safety of dasiglucagon in children with CHI was conducted at 11 sites in 4 countries (USA, UK, Germany, and Israel) between January 7, 2019, and October 5, 2020. Patients were randomly assigned to continue receiving SoC alone, or SoC plus dasiglucagon (10-70 µg/h via an infusion pump) for 4 weeks (Part 1). In Part 2, all patients received SoC plus dasiglucagon for 4 weeks (Fig. 1). Participants completing the trial were given the opportunity to continue dasiglucagon treatment in an ongoing long-term extension trial (ZP4207-17106; www.clinicaltrials.gov NCT03941236) conditional on investigator confirmation of a continued positive benefit-risk balance. Patients not continuing into the extension trial attended a follow-up visit 4 weeks after stopping dasiglucagon treatment.

The trial protocol, consent form, and other information provided to participants and parents/legal representatives were approved by independent ethics committees or institutional review boards at participating sites, and by competent authorities. The trial was conducted according to the Declaration of Helsinki and Good Clinical Practice with written informed consent obtained from parents/caregivers and assent from participants (as required) before trial enrollment. The trial is registered with www.clinicaltrials.gov (NCT03777176).

### Participants

Eligible participants were aged between 3 months and 12 years (both inclusive), had a body weight ≥4 kg, a documented diagnosis of CHI, and were experiencing ≥3 episodes of hypoglycemia/week (self-measured plasma glucose [SMPG] < 3.9 mmol/L) according to the investigator's evaluation. Patients could either have undergone near-total pancreatectomy (>95%) or were treated nonsurgically. Patients requiring exogenous insulin, or who had documented glycated hemoglobin (HbA_1c_) ≥ 7% (53 mmol/mol) after near-total pancreatectomy (and within 6 months prior to screening) were excluded. Patients were also excluded if there were significant changes to SoC medications during screening or they had received glucagon in the 24 hours prior to randomization. Concurrent use of glucagon and dasiglucagon was not permitted during the trial. Doses of all SoC medications were to remain stable throughout the trial. If the participant was receiving octreotide or lanreotide, these must have been used for at least 8 and 16 weeks prior to screening, respectively.

### Randomization

Patients were randomly assigned (using an interactive web response system) in a 1:1 ratio using a block randomization scheme stratified by region (USA/non-USA) to continue receiving SoC alone, or SoC plus dasiglucagon for 4 weeks (Part 1). In Part 2, all patients received SoC plus dasiglucagon for 4 weeks (Fig. 1). Stratification addressed regional differences in CHI treatment (eg, differences between North America and Europe in the frequency of subtotal pancreatectomy (≤95%) for diffuse CHI). The trial was open-label apart from continuous glucose monitoring (CGM) results which were blinded to all patients, parents/caregivers, and trial personnel until after database lock.

### Procedures

Following screening, eligible patients underwent a 2-week blinded CGM period using a Dexcom G4 device (Dexcom Inc., San Diego, CA, USA) to establish baseline glycemic profiles. This period was also used to confirm eligibility criteria frequency of ≥3 episodes of hypoglycemia (SMPG <3.9 mmol/L) per week via SMPG measurements using a StatStrip Xpress 2 glucose meter (Nova Biomedical, Waltham, MA, USA).

At the beginning of Part 1 (Week 1), all patients were admitted as inpatients for 1 to 2 days. Patients assigned to SoC plus dasiglucagon treatment received dasiglucagon infusion (4 mg/mL: Zealand Pharma, Søborg, Denmark) via an Accu-Chek Spirit Combo infusion pump (Hoffman-La Roche AG, Basel, Switzerland). Two types of infusion sets could be used that had either a steel or a Teflon needle. Primarily, the infusion set with the Teflon needle was used. The steel infusion set was changed every second day, and the Teflon infusion set was changed every third day. Infusion was initiated at 10 μg/h, with the dose increased by 10 μg/h every 2 hours to a maximum rate of 70 μg/h unless the patient was weaned off gastric dextrose infusion and/or glucose fortified feeds, plasma glucose levels during the previous 2 hours were consistently >6.7 mmol/L, or adverse events considered related to dasiglucagon (eg, nausea and vomiting) limited further dose escalation. The dose increase in dasiglucagon was restricted to achieve the treatment objectives of obtaining a SMPG value between 3.9 and 6.7 mmol/L while approaching a normal feeding regimen according to age.

In Part 2, patients originally assigned to receive SoC alone in Part 1 were admitted as inpatients for the first 1 to 2 days of Part 2 (Week 5) and had dasiglucagon infusion initiated and titrated as described above. Regardless of whether dasiglucagon was initiated during Part 1 or 2, SoC medications for CHI were to be kept constant throughout the trial. Adjustments to gastric and oral feeds were permitted.

All patients and/or their parent(s)/guardian were trained and supervised in the use of the infusion pump, CGM device, and handheld glucose meter, with SMPG measurements recorded in a patient diary. Each patient was required to have a SMPG measurement at least 3 times/day (preferably before main meals) and when hypoglycemia was suspected. CGM was used in a blinded manner, with glucose measurements not available to patients or investigators during the study period. The blinded CGM device was worn by patients during the entire trial including the 2-week period prior to randomization. Short periods of CGM discontinuation for 1 to 3 days were allowed on investigator discretion in the event of skin irritation, discomfort, or other valid reasons.

### Outcomes

The primary endpoint was the rate of SMPG-detected hypoglycemia (average weekly number of episodes of SMPG <3.9 mmol/L) during Weeks 2 to 4.

There were 3 key secondary efficacy endpoints: increase in fasting tolerance (time from beginning of meal to the beginning of the first continuous 15-minute CGM reading <3.9 mmol/L), CGM-detected percent time in range (3.9-10.0 mmol/L) during Weeks 2 to 4, and rate of clinically significant SMPG-detected hypoglycemia (average weekly number of episodes of SMPG <3.0 mmol/L) during Weeks 2 to 4. Other secondary efficacy endpoints included rate of SMPG readings per week during Weeks 2 to 4, total amount of gastric carbohydrates administered via nasogastric (NG) tube or gastrostomy tube per week during Weeks 2 to 4, and the following measures of CGM-detected hypoglycemia during Weeks 2 to 4: average weekly rate of overall hypoglycemia (<3.9 mmol/L), average weekly percent time in hypoglycemia (<3.9 mmol/L), and average weekly extent of hypoglycemia (<3.9 mmol/L and <3.0 mmol/L).

Safety endpoints included adverse events (AEs), changes in clinical evaluations of vital signs, physical examination, and 12-lead electrocardiograms, and changes in laboratory assessments of hematology, biochemistry, and anti-drug antibodies (ADAs). ADAs assessed were Abcam Cat# ab92517, RRID:AB_10561971 and Zealand Pharma Cat# Mab5438-M-F1-3, RRID:AB_3073520 (www.antibodyregistry.org).

### Statistical Analyses

The trial was powered to detect a treatment effect of 50% for the primary endpoint, under the assumption that patients randomized to SoC alone maintained a similar rate of hypoglycemia to baseline in Weeks 2 to 4 that followed a Poisson distribution with a mean of 9, whereas the number of hypoglycemia events reported for patients in the dasiglucagon group during Weeks 2 to 4 followed a Poisson distribution with a mean of 4.5. A sample size of 32 patients was estimated to have 99% power testing at a 0.05 significance level. The primary endpoint was analyzed using negative binomial regression, with region and treatment group as fixed effects, baseline hypoglycemia rate (average weekly number of hypoglycemia episodes [SMPG <3.9 mmol/L] during the 2-week baseline period) as covariate, and number of hypoglycemia episodes (SMPG <3.9 mmol/L) during Weeks 2 to 4 as the dependent variable. Log-transformed number of weeks in Weeks 2 to 4 of Part 1, after accounting for imputation (ie, log[3] for patients with imputed data, or number of weeks in Weeks 2-4 otherwise), was used as an offset variable. The null hypothesis was no difference in the average weekly number of SMPG measured hypoglycemia episodes between the 2 treatment groups (α = .05). The primary analysis estimated the treatment effect based on the de facto (treatment policy) estimand. All available data in the form of actual measurements were therefore included in the analysis, irrespective of adherence to treatment or use of subsequent therapy.

The rate of clinically significant SMPG-detected hypoglycemia (SMPG <3.0 mmol/L) was analyzed as for the primary endpoint. We used analysis of covariance (ANCOVA) to analyze increases in fasting tolerance and CGM-detected percent time in range (where percent time was calculated as number of minutes in range/total number of minutes patient was wearing CGM) per week during Weeks 2 to 4, with treatment group and region as fixed effects, and baseline values as covariates. ANCOVA was also used to analyze total amount of gastric carbohydrates administered via NG tube or gastrostomy (post hoc analysis).

In addition to SMPG, CGM-detected hypoglycemia frequencies were analyzed (post hoc) as for the primary endpoint. A single CGM-detected hypoglycemia episode was defined as CGM <3.9 mmol/L or <3.0 mmol/L for 15 minutes (18) and up to 60 minutes from onset, even if normoglycemia (>3.9 mmol/L) was not reached within this time. A new episode of hypoglycemia was reported when the next CGM value was below 3.9 mmol/L (or <3.0 mmol/L) for at least 15 minutes (18). CGM-detected percent time in hypoglycemia (defined as percent time below 3.9 mmol/L or 3.0 mmol/L) was analyzed using a generalized linear model assuming a normal distribution and a log link function, with region and treatment group as fixed effects and log (baseline time in hypoglycemia) as covariate (post hoc analysis). CGM-detected extent of hypoglycemia (defined as area over glucose curve under 3.9 mmol/L or 3.0 mmol/L divided by total duration of CGM measurement) was analyzed using ANCOVA using region and treatment group as fixed effects and baseline extent of hypoglycemia as covariate (post hoc analysis).

The overall type 1 error was controlled by adopting a hierarchical (fixed sequence) testing procedure for the primary and key secondary endpoints (Supplementary Table S1 (19)). Statistical analyses were run using SAS Version 9.4 (SAS Institute, Cary, NC, USA).

## Results

In total, 35 patients were screened, and 32 were enrolled and randomly assigned to treatment (dasiglucagon + SoC: n = 16; SoC: n = 16), all of whom completed Part 1 and entered Part 2 of the trial. Dasiglucagon treatment was subsequently prematurely discontinued in 1 patient due to hyperglycemia and ketosis (clinical evaluation did not confirm diabetic ketoacidosis). All 32 patients were included in the full analysis and safety analysis sets (Supplementary Fig. S1 (19)). Thirty-one of 32 patients continued dasiglucagon treatment in a long-term extension trial following investigator confirmation of a continued positive benefit-risk balance.

Overall, treatment groups were comparable with respect to demographic and baseline characteristics. At entry, the trial population had a mean age of 4.3 years (range, 0.6-10.9 years), a mean weight of 19.9 kg (range. 7.9-59.8 kg), and 50% of participants were female. Most participants were White (22/32), and approximately 30% of participants (11/32) had undergone subtotal or near-total pancreatectomy prior to the trial (Table 1).

**Table 1.** Baseline and demographic characteristics

	Randomized treatment		Overall
	Dasi + SoC	SoC only	
Number of patients (n)	16	16	32
Age, years	3.6 (2.6) Range, 0.6-10.8	5.0 (2.9) Range, 1.5-10.9	4.3 (2.8) Range, 0.6-10.9
Male/Female (n)	10/6	6/10	16/16
Height, cm	94.8 (18.8)	105.6 (20.5)	100.2 (20.1)
Weight, kg	17.2 (6.9)	22.8 (12.7)	19.9 (10.5)
Weight Z-score	0.74 (1.73)	0.82 (1.41)	0.78 (1.55)
Pancreatectomy (n)			
None	12	9	21
Subtotal (≤95%)	3	4	7
Near total (>95%)	1	3	4
Gastrostomy/NG tube (n)			
None	5	3	8
Gastrostomy	9	12	21
NG tube	2	1	3
Standard of care medications (n)	16	15	31
Diazoxide	6	4	10
Somatostatin analogues	9	11	20
Glucagon	5	3	8
Sirolimus	0	1	1

Values are mean (SD) unless otherwise specified; weight Z-scores (based on the World Health Organization growth charts) were derived using a subject's age and sex; concurrent use of glucagon and dasiglucagon was not permitted during the trial. Patients were excluded from participation if they had used glucagon within 24 hours prior to randomization. Abbreviations: Dasi, Dasiglucagon; n, number of patients; NG, nasogastric; SoC, standard of care.

**Figure 2.**
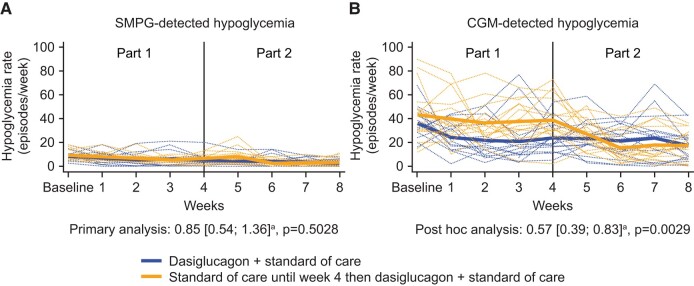
Individual and mean SMPG- vs CGM-detected hypoglycemia episodes/week (PG <3.9 mmol/L). Notes: Thick lines are mean profiles; narrow dotted lines are individual patient profiles. Abbreviations: CGM: continuous glucose monitoring; SMPG: self-measured plasma glucose; ^a^ Event rate ratio (dasiglucagon + standard of care/standard of care alone) during Weeks 2 to 4.

The rate of SMPG-detected hypoglycemia episodes (SMPG <3.9 mmol/L) decreased from baseline in both treatment groups, but no statistically significant difference was found between dasiglucagon + SoC vs SoC alone during Weeks 2 to 4 (primary analysis: mean [95% CI] 0.85 [0.54; 1.36], *P* = .5028; Fig. 2, Panel A and Table 2). Of note, patients randomized to dasiglucagon + SoC had a 25% higher median rate of weekly SMPG readings in Weeks 2 to 4 than those in the SoC-only group (45 vs 36 readings, respectively).

**Table 2.** Hypoglycemia event rates

Measure	Mean number of hypoglycemia events/week				Event rate ratio*^a^* [95% CI] (Weeks 2-4: Dasi + SoC/SoC)
	Dasi + SoC (Baseline) n = 16	Dasi + SoC (Weeks 2-4) n = 16	SoC (Baseline) n = 16	SoC (Weeks 2-4) n = 16	
SMPG <3.9 mmol/L	8.3	5.3	9.0	5.9	0.85 [0.54; 1.36], *P* = .5028*^b^*
CGM <3.9 mmol/L*^c^*	36.0*^d^*	22.0	43.0	37.0	0.57 [0.39; 0.83], *P* = .0029*^e^*
SMPG <3.0 mmol/L	2.3	1.8	2.3	1.9	0.93 [0.49; 1.74], *P* = .8114*^f^*
CGM <3.0 mmol/L*^c^*	13.9*^d^*	8.5	18.2	14.0	0.56 [0.37; 0.86], *P* = .0075*^e^*

Abbreviations: CGM, continuous glucose monitoring; Dasi, dasiglucagon; n, number of patients; SMPG, self-measured plasma glucose; SoC, standard of care.

^*a*^Estimate of event rate ratio.

^*b*^Primary analysis.

^*c*^A single CGM-detected hypoglycemia episode is defined as CGM <3.9 mmol/L (or <3.0 mmol/L) for 15 minutes and up until 60 minutes from the start of the episode even if normoglycemia (>3.9 mmol/L) is not reached within this time. A new episode of hypoglycemia is defined as when the next CGM value was below 3.9 mmol/L (or <3.0 mmol/L) for at least 15 minutes.

^*d*^n = 15.

^*e*^Post hoc analysis.

^*f*^Key secondary analysis.

In line with the primary endpoint, no statistically significant difference was found between treatment groups with respect to rates of clinically significant SMPG-detected hypoglycemia (SMPG <3.0 mmol/L; Table 2). However, since the primary endpoint was not met, results for clinically significant SMPG-detected hypoglycemia and the other 2 key secondary endpoints in the statistical testing hierarchy need to be interpreted accordingly.

In contrast to the SMPG-derived data, dasiglucagon treatment resulted in a 43% reduction in CGM-detected hypoglycemia (<3.9 mmol/L) compared to SoC alone during Weeks 2 to 4 (post hoc analysis: mean [95% CI] 0.57 [0.39; 0.83], *P* = .0029; Fig. 2, Panel B and Table 2). Likewise, CGM-detected clinically significant hypoglycemia (<3.0 mmol/L) was reduced by a similar magnitude with dasiglucagon treatment (Table 2). Dasiglucagon was also associated with consistent reductions (of 37% to 61%) in all other measures of hypoglycemia assessed by blinded CGM vs SoC alone including nocturnal hypoglycemia (post hoc analyses; Table 3). Improvements in hypoglycemia rates were also observed in the patients switching from SoC alone to dasiglucagon + SoC in Part 2 (Fig. 2).

**Table 3.** CGM measures of hypoglycemia

Measure	Mean observed values				Ratio*^a^* (Weeks 2-4: Dasi + SoC/SoC)
	Dasi + SoC (Baseline) n = 15	Dasi + SoC (Weeks 2-4) n = 16	SoC (Baseline) n = 16	SoC (Weeks 2-4) n = 16	
% time <3.9 mmol/L	20.7	10.8	22.0	20.2	0.53 [0.36; 0.79], *P* = .0017*^b^*
% time <3.0 mmol/L	5.9	3.1	7.4	6.3	0.49 [0.30; 0.82], *P* = .0061*^b^*
Extent of hypoglycemia <3.9 mmol/L*^c^*	0.14	0.08	0.17	0.15	0.49 [0.31; 0.78], *P* = .0038*^d^*
Extent of hypoglycemia <3.0 mmol/L*^c^*	0.02	0.02	0.03	0.03	0.55 [0.33; 0.90], *P* = .0207*^d^*
Nocturnal hypoglycemia <3.9 mmol/L*^e^*	11.9	5.9	11.8	9.5	0.39 [0.21; 0.75], *P* = .0047*^f^*
Nocturnal hypoglycemia <3.0 mmol/L*^e^*	3.9	2.0	4.9	3.5	0.63 [0.32; 1.24], *P* = .1775*^f^*

Abbreviations: Dasi, dasiglucagon; SoC, standard of care; CGM, continuous glucose monitoring.

^*a*^Post hoc analyses.

^*b*^Estimate of least squares (LS) means ratio.

^*c*^Extent of hypoglycemia is defined as area over glucose curve under 3.9 mmol/L (or 3.0 mmol/L) as measured by CGM divided by total duration in hours of CGM measurement.

^*d*^Estimate of geometric mean ratio.

^*e*^Mean number of nocturnal hypoglycemia events/week (events with onset between 10 Pm [excluded] and 6 Am [excluded]); a single CGM-detected hypoglycemia episode is defined as CGM <3.9 mmol/L (or <3.0 mmol/L) for 15 minutes and up until 60 minutes from the start of the episode even if normoglycemia (>3.9 mmol/L) is not reached within this time. A new episode of hypoglycemia is defined as when the next CGM value was below 3.9 mmol/L (or <3.0 mmol/L) for at least 15 minutes.

^*f*^Estimate of event rate ratio.

In total, 24 of the 32 trial patients received carbohydrates via NG tube or gastrostomy: 11 patients in the dasiglucagon + SoC group and 13 patients in the SoC-only group. Despite large variation between the patients with respect to the total amount of gastric carbohydrate administered via NG tube or gastrostomy, the mean total weekly gastric carbohydrate intake decreased from baseline for the dasiglucagon + SoC (−146.56 g) but increased for patients receiving SoC alone (+160.44 g) during Weeks 2 to 4 (Supplementary Fig. S2 (19)). A post hoc analysis showed a trend toward a treatment difference (−253 g/week [−563; 58] *P* = .105). Consistent with this, in Part 2 of the trial, mean total gastric carbohydrate intake also decreased for patients previously on SoC alone upon initiation of dasiglucagon treatment (Supplementary Fig. S2 (19)).

The mean weekly dasiglucagon infusion rate was 30.1 μg/h at the end of Part 1 of the trial (n = 16) and 34.3 μg/h after Part 2 (n = 32). A minority of patients (8 of 32) were up-titrated to the maximum infusion rate of 70 μg/h (Supplementary Fig. S3 (19)). There was no impact of age, body weight, or severity of CHI based on baseline hypoglycemia (number of SMPG-detected events [range, 2.5-18] or percentage of time <3.9 mmol/L measured by CGM [range, 3.8%-47.9%]) on dasiglucagon dose (Supplementary Figs. S4-7 (19)).

No statistically significant difference was found between treatment groups with respect to the key secondary endpoint of CGM-detected percent time in normal glucose range (3.9-10 mmol/L) during Weeks 2 to 4 (treatment difference: 0.15% [−6.5%; 6.8%], *P* = .9653). However, post hoc analyses showed that during Weeks 2 to 4, CGM-detected percent time <3.9 mmol/L was lower for dasiglucagon + SoC vs SoC alone (treatment ratio: 0.53 [0.36; 0.79], *P* = .0017; Table 2) whereas percent time >10 mmol/L was higher (treatment ratio: 2.44 [1.30; 4.47], *P* = .0055). For the remaining key secondary endpoint, fasting tolerance, several procedural issues with the fasting tolerance test precluded a meaningful interpretation of the results.

Over the course of the trial, 6 serious AEs (vascular device infection, localized infection, folliculitis, H1N1 influenza, hypoglycemia, and hyperglycemia) were reported for 4 patients; no event was considered related to dasiglucagon treatment by the investigator. During Part 1 of the trial, more AEs were reported in the dasiglucagon + SoC group than with SoC alone. This imbalance was largely accounted for by AEs in the system organ classes “Gastrointestinal disorders,” “Infections and infestations,” and “Skin and subcutaneous tissue disorders” (Table 4), the majority of which were mild in severity. The most frequent gastrointestinal disorder was vomiting, a common side effect of glucagon treatment. There was no obvious pattern in the severity or type of infection and infestation–related AEs; none were considered related to dasiglucagon treatment and, apart from upper respiratory tract infection and eczema (infected), none were reported in more than 1 patient per treatment group. Five skin and subcutaneous tissue disorder-related AEs were reported as suspicion of necrolytic migratory erythema, of which 2 were subsequently clinically confirmed (both for patients randomized to SoC alone in Part 1 of the trial, and with onset in Part 2) by a dermatologist and the principal investigator at that site. Both cases were nonserious, with 1 case mild and the other moderate in severity. Both cases were considered possibly or probably related to dasiglucagon; both events were manageable by dose adjustment with neither leading to a discontinuation of dosing. There were 2 reports of needle occlusion, neither of which were related to any AEs or episodes of hypoglycemia. No noteworthy abnormalities were found in the biochemistry, hematology, vital signs, electrocardiography, echocardiography, and neurological assessments following dasiglucagon treatment. Clinically significant increases in aspartate aminotransferase (AST) were noted for 3 patients in the dasiglucagon + SoC group, 2 of whom also had clinically significant increases in alanine aminotransferase (ALT); all cases were assessed as not/unlikely related to dasiglucagon, and no patient fulfilled the criteria of Hy's law. Among the 30 children with anti-drug antibody (ADA) assessments, 7/30 (23.3%) were ADA-positive during the trial: 1 patient had treatment-boosted and 6 treatment-induced antibodies. Of these 7 children, 1 child had cross-reactivity to glucagon and another child had in vitro neutralizing antibodies to dasiglucagon at the follow-up visit. In vitro neutralizing antibodies were not detected for the remaining 5 children with ADA.

**Table 4.** Summary of commonly reported treatment-emergent adverse events

	Part 1		Part 2	Part 1 + 2
System Organ Class Preferred Term	Dasi + SoC (n = 16)	SoC (n = 16)	Dasi + SoC (n = 32)	Dasi + SoC (n = 32)
	n (%) E	n (%) E	n (%) E	n (%) E
**Patients with at least 1 TEAE**	**14 (87.50%) 50**	**8 (50.00%) 11**	**24 (75.00%) 78**	**27 (84.38%) 128**
**Total duration of exposure*^a^***	**1.2**	**1.3**	**2.6**	**3.8**
**Infections and Infestations**	**9 (56.25%) 12**	**4 (25.00%) 4**	**9 (28.13%) 12**	**15 (46.88%) 24**
** Upper Respiratory Tract Infection**	**2 (12.50%) 2**	**0 (0.00%) 0**	**2 (6.25%) 2**	**4 (12.50%) 4**
** Eczema Infected**	**0 (0.00%) 0**	**0 (0.00%) 0**	**3 (9.38%) 3**	**3 (9.38%) 3**
**Skin and Subcutaneous Tissue Disorders**	**9 (56.25%) 14**	**0 (0.00%) 0**	**10 (31.25%) 14**	**16 (50.00%) 28**
** Eczema**	**5 (31.25%) 5**	**0 (0.00%) 0**	**1 (3.13%) 1**	**6 (18.75%) 6**
** Rash**	**3 (18.75%) 4**	**0 (0.00%) 0**	**2 (6.25%) 2**	**5 (15.63%) 6**
** Rash Maculo-Papular**	**1 (6.25%) 1**	**0 (0.00%) 0**	**2 (6.25%) 2**	**3 (9.38%) 3**
**Gastrointestinal Disorders**	**6 (37.50%) 14**	**2 (12.50%) 2**	**5 (15.63%) 9**	**10 (31.25%) 23**
** Vomiting**	**5 (31.25%) 7**	**1 (6.25%) 1**	**2 (6.25%) 5**	**7 (21.88%) 12**
** Diarrhea**	**2 (12.50%) 2**	**1 (6.25%) 1**	**0 (0.00%) 0**	**2 (6.25%) 2**
** Teething**	**2 (12.50%) 2**	**0 (0.00%) 0**	**0 (0.00%) 0**	**2 (6.25%) 2**
**Metabolism and Nutrition Disorders**	**0 (0.00%) 0**	**1 (6.25%) 1**	**6 (18.75%) 21**	**6 (18.75%) 21**
** Hyperglycemia**	**0 (0.00%) 0**	**0 (0.00%) 0**	**5 (15.63%) 17**	**5 (15.63%) 17**

Commonly reported treatment-emergent adverse events (TEAEs) are TEAEs reported by at least 2 patients in either treatment group in Part 1 of the trial or in at least 3 patients receiving dasiglucagon throughout the trial. Percentages are calculated as n/total number of patients in the group multiplied by 100. Patients are summarized by treatment received at the start of AE. Part 1 + 2: Dasiglucagon + SoC (Part 1) and Dasiglucagon + SoC (Part 2) combined.

Abbreviations: AE, adverse event; Dasi, dasiglucagon; E, number of events; n, number of patients; SoC, standard of care; TEAE, treatment-emergent AE defined as events that began after the first dose of dasiglucagon (dasiglucagon + SoC treatment group) or following randomization (SoC-only treatment group).

^*a*^Total duration of exposure: total time of exposure to SoC or to dasiglucagon + SoC, as applicable.

## Discussion

This 2-arm, open-label, randomized trial investigated the efficacy and safety of dasiglucagon administered via SC continuous infusion on top of standard care for the treatment of children with CHI aged between 3 months and 12 years.

The results of the prespecified primary endpoint analysis showed no statistically significant difference between treatment groups in the weekly rate of SMPG-detected hypoglycemia (SMPG <3.9 mmol/L) after 4 weeks of treatment. Importantly, post hoc analyses showed that dasiglucagon treatment resulted in consistent, clinically meaningful reductions in all measures of hypoglycemia assessed by blinded CGM compared to SoC alone during this period. These findings applied to both hypoglycemia of <3.9 mmol/L and clinically significant hypoglycemia (<3.0 mmol/L).

Primary and secondary hypoglycemia endpoints were chosen with reference to regulatory expectations, and in collaboration with specialist CHI treatment centers and patient organizations, taking into consideration that CHI is a rare disease in which data and outcome measure validation are sparse and the threshold for hypoglycemia is not clearly defined. For hypoglycemia, SMPG was used as the primary endpoint, while CGM was used for several secondary endpoints, albeit off-label with respect to both indication and age range (20).

The failure to detect a treatment difference with SMPG in our trial may be partly attributable to differences in the way information on hypoglycemia is captured with SMPG measurements compared to blinded CGM. Blood glucose meters (used for SMPG) provide information about discrete, intermittent blood glucose levels and therefore, unless performed very frequently, may not capture all episodes of hypoglycemia, especially during periods when patients are unable to measure their blood glucose or are hypoglycemia-symptom unaware. In addition, the unblinded glucose measurements obtained with SMPG may have resulted in changes in patient behavior (eg, additional food intake, change in timing of meals). In contrast, measurements every 5 minutes (up to 288 measurements daily) obtained using blinded CGM may provide a greater likelihood of capturing episodes of asymptomatic or nocturnal hypoglycemia. Despite concerns over the absolute accuracy of CGM, particularly in lower glucose ranges, the continuous nature of CGM provides a more comprehensive glycemic profile, including time spent in hypoglycemia, in hyperglycemia, and within the euglycemic range. During Weeks 2 to 4, subjects randomized to dasiglucagon + SoC reported a 25% higher rate of weekly SMPG readings. Consequently, the potentially greater effectiveness of hypoglycemia detection in this group relative to the SoC-only group may have introduced detection bias to the disadvantage of the dasiglucagon + SoC group.

The reduction in CGM-detected hypoglycemia with dasiglucagon was observed concurrent to a reduction from baseline of ∼150 g in mean weekly total gastric carbohydrate administration via NG tube or gastronomy in the dasiglucagon + SoC group during Weeks 2 to 4. In contrast, patients randomized to SoC alone tended to maintain or increase their carbohydrate intake during this period (mean weekly increase of ∼150 g). These observations suggest that treatment with dasiglucagon reduces the need for carbohydrate intake to avoid hypoglycemia. Consistent with this, in Part 2 of the trial, mean total gastric carbohydrate intake also decreased for patients previously on SoC alone.

CGM demonstrated a dasiglucagon-associated reduction in the rate of overall and nocturnal hypoglycemia, where the risk of nocturnal hypoglycemia has been shown to be 2 to 3 times higher than at other times of the day (21). CGM also revealed clinically meaningful benefits of dasiglucagon on both the percent time spent in hypoglycemia and extent of hypoglycemia, endpoints that cannot be measured using intermittent SMPG. Such information is particularly valuable for a child with CHI because, in addition to the number of discrete episodes of hypoglycemia experienced, a reduction in the extent and length of time spent in hypoglycemia is also likely to be protective of childhood neurodevelopment.

A dasiglucagon dose range of 10 to 70 μg/h was chosen for the trial based on clinical experience from the off-label use of glucagon in CHI patients (9, 14) and supportive data from a pharmacokinetic/pharmacodynamic model developed for a pediatric population (>25 kg) with type 1 diabetes (unpublished). We utilized the full dose range to treat patients according to their individual needs in our trial, with the investigated dose range appearing to be suitable for a wide spectrum of CHI patients; there was no indication that body weight, age, or severity of CHI (based on baseline hypoglycemia) had an impact on the required dasiglucagon dose; instead, dasiglucagon dose was determined by individual response.

Dasiglucagon treatment was generally safe and well tolerated in the study. More cases of skin and subcutaneous tissue disorders were reported for dasiglucagon relative to SoC treatment, including 2 clinically confirmed cases of necrolytic migratory erythema, a skin condition that may arise from sustained exposure to high levels of glucagon (22). However, no skin-related events led to discontinuation of dosing. Vomiting was reported more frequently in the dasiglucagon + SoC treatment group compared with SoC only, consistent with gastrointestinal events being well-known side effects of glucagon treatment.

To our knowledge, our trial of 32 patients is the largest randomized, controlled study of glucagon or glucagon analogues for the treatment of CHI conducted to date. Until now, investigations into the use of glucagon for the prevention and treatment of hypoglycemia in CHI have been limited to short-term observational studies and case reports of the intermittent IV administration of human glucagon (9, 10, 14), and an ongoing phase 2 study investigating once-weekly SC administration of a glucagon analogue, HM15136 (23). A potential weakness of our study was that the selected trial population and trial procedures did not cover the extent of heterogeneity of clinical management preferences among all international centers treating CHI patients. To ensure uniformity of study design and data, procedures had to be undertaken that deviated from standard clinical care. For example, the need to keep baseline doses and type of SoC medications constant throughout the study (albeit not adhered to by all patients) to allow an optimal comparison between the treatment groups may have led to more events of hypoglycemia and hyperglycemia, even though adjustments to gastric and oral feeding were permitted. Another possible weakness of the trial was that the time and frequency of SMPG measurements were not fixed to prevent potential differences between treatment groups that could bias the treatment effect estimate for the primary endpoint. Due to the nature of the disease, it was not possible to control carbohydrate intake via NG tube or gastronomy for the purposes of the trial. Consequently, the greater carbohydrate intake observed for the SoC-only group is likely to have reduced treatment differences with respect to hypoglycemia.

In conclusion, our large international trial involving major CHI treatment centers demonstrated that dasiglucagon treatment was associated with marked, clinically meaningful reductions in all measures of hypoglycemia recorded by blinded CGM. It was also noteworthy that 31 of 32 patients who completed the trial continued dasiglucagon treatment in a long-term extension trial following investigator confirmation of a continued positive benefit-risk balance. Pending the results of this extension trial, dasiglucagon may therefore represent an attractive new therapeutic option for the prevention and treatment of hypoglycemia in CHI. Should this be realized, dasiglucagon treatment could have the potential to reduce the detrimental effects of hypoglycemia on childhood neurodevelopment and to delay and ultimately avoid pancreatectomy and related exocrine and endocrine complications.

## Data Availability

Restrictions apply to the availability of some or all data generated or analyzed during this study to preserve patient confidentiality or because they were used under license. The corresponding author will on request detail the restrictions and any conditions under which access to some data may be provided.

## Clinical Trial Information

Clinical trial ID: ZP4207-17106; NCT no. NCT03941236 (www.clinicaltrials.gov).

## Abbreviations

ADA: anti-drug antibody
AE: adverse event
ANCOVA: analysis of covariance
CGM: continuous glucose monitoring
CHI: congenital hyperinsulinism
IV: intravenous
NG: nasogastric
SC: subcutaneous
SMPG: self-monitored plasma glucose
SoC: standard of care
